# Supplementary material for: Healthy, Inclusive and Sustainable Catering in Secondary Schools—An Analysis of a Transformation Process with Multiple Tensions
Source: Int J Environ Res Public Health. 2024 Mar 20;21(3):370. doi: 10.3390/ijerph21030370 (PMC10969783; doi:10.3390/ijerph21030370)
Supplement: Supplementary file 1 [file ijerph-21-00370-s001.zip › ijerph-2803757-supplementary.pdf]

TU Berlin I ZTG I Sekr. KAI 3-2 | Kaiserin-Augusta-Allee 104 | 10553 Berlin | *www.tu-berlin.de/ztg*

Supplementary Table S1 Survey Form. Healthy, Inclusive and Sustainable Catering in Secondary Schools

We would like to ask you a few introductory questions about yourself.	1.1 What year were you born?	
	1.2 What is your gender?	☐ female<br>☐ male<br>☐ diverse
	1.3 What is your school?	Please select
	1.4 What grade level are you in?	☐ 8<br>☐ 9<br>☐ 10
2. Now we would like to ask you a few questions about the food at your school.	2.1 How often do you eat warm lunch at school?	☐ Daily<br>☐ 3-4 times per week<br>☐ 1-2 times per week<br>☐ Less often<br>☐ Never<br>☐ Not available
	2.2 Why do you never or rarely eat at school? Please select all the answers that apply.	☐ Because I prefer to eat dinner at home.<br>☐ Because I do not like the taste of the food.<br>☐ Because the price is too high for me and my parents.<br>☐ Because the lunchbreak is too short.<br>☐ Because I prefer to eat with my friends outside of school.<br>☐ Because I prefer to buy something from the kiosk/vending machine.<br>☐ Because the lunchroom is not appealing<br>☐ Because of little variety in food/small menu.<br>☐ Because the food contains too many calories and fat.<br>☐ Because my friends do not eat at school.

		☐ Because I bring my food from home.<br>☐ Because I feel overly supervised by teachers.<br>☐ Because the food does not fit into my personal diet.<br>☐ Because I am not particularly hungry at noon.<br>☐ Because the portion size is too small<br>☐ Because the portion size is too large<br>☐ Other reasons: …
	2.3 Which of the following food options are available in addition to or instead of the warm lunch at your school? Please select all the answers that apply.	☐ Kiosk<br>☐ After-lunch offer in the canteen<br>☐ Vending machines<br>☐ No other food options available
	2.4 How often do you get small snacks there?	☐ Daily<br>☐ 3-4 times per week<br>☐ 1-2 times per week<br>☐ Less often<br>☐ Never
	2.5 What exactly are you buying?	

	Daily	3-4 times per week	1-2 times per week	Less often	Never
Sandwiches/bread with sausage or cheese	☐	☐	☐	☐	☐
Sweet pastries (croissants, donuts, or muffins)	☐	☐	☐	☐	☐
Sweet or salty snacks	☐	☐	☐	☐	☐
Dairy products such as yoghurt, quark	☐	☐	☐	☐	☐

		warm snacks such as schnitzel, meatballs, pizza slices, hot sausages	☐	☐	☐	☐	☐
		Fruit or fruit salad	☐	☐	☐	☐	☐
		Raw vegetables	☐	☐	☐	☐	☐
		Sweet drinks (Cola, lemonade, energy drinks or ice tea)	☐	☐	☐	☐	☐
	2.6 How often do you eat nothing at all for lunch?	☐ Daily<br>☐ 3-4 times per week<br>☐ 1-2 times per week<br>☐ Less often ☐ Never					
3. Jetzt würden wir gerne noch etwas über den Ablauf des Schulmittagessens erfahren.	3.1. Which of the following statements apply to you eating school lunch?		Agree	Rather agree	Rather disagree	Disagree	
		We eat lunch together with the class	☐	☐	☐	☐	
		I only eat lunch with my friends	☐	☐	☐	☐	
		The school lunch is an opportunity for me to talk to other pupils that I do not know so well.	☐	☐	☐	☐	
	3.2 Do pupils have to pre-order the food?	☐ Yes<br>☐ No<br>☐ I do not know					
	3.3. At your school, how long in advance do you usually have to place your food order?	☐ ____ Days<br>☐ I do not know					

	3.4 What applies to the food service?	☐ We are served a complete meal at the counter.<br>☐ We can select at least some of the food components by ourselves<br>☐ Food is served differently: ___<br>☐ I do not know
	3.5 How much time do you have for lunch at your school?	☐ Less than 20 minutes<br>☐ 20-30 minutes<br>☐ 31-45 minutes<br>☐ 46-60 minutes<br>☐ More than one hour
4. In this section, we would like to know how you rate the lunch and what your preferences are.	4.1 How satisfied are you with the following aspects of the school lunch?	(see table below)

	1 = not satisfied	2	3	4	5 = very satisfied
Taste	☐	☐	☐	☐	☐
Temperature	☐	☐	☐	☐	☐
Food presentation	☐	☐	☐	☐	☐
Menu	☐	☐	☐	☐	☐
Length of lunchbreak	☐	☐	☐	☐	☐
Meal ordering system	☐	☐	☐	☐	☐
Service workers	☐	☐	☐	☐	☐

	4.2 How satisfied are you with the following aspects of the lunchroom?		1 = not satisfied	2	3	4	5 = very satisfied
		Noise level	☐	☐	☐	☐	☐
		Available seating	☐	☐	☐	☐	☐
		Cleanliness	☐	☐	☐	☐	☐
		Comfort	☐	☐	☐	☐	☐
	4.3 How important is it to you that the school lunch offers the following aspects?		1 = Not important	2	3	4	5 = Very important
		Organic food	☐	☐	☐	☐	☐
		Healthy meals	☐	☐	☐	☐	☐
		Faire trade products	☐	☐	☐	☐	☐
		Vegetarian dishes	☐	☐	☐	☐	☐
		Vegan dishes	☐	☐	☐	☐	☐
		Regional food	☐	☐	☐	☐	☐
		Information about the origins of the ingredients	☐	☐	☐	☐	☐
		Comfortable lunchroom	☐	☐	☐	☐	☐

		Opportunities to decide about the offered menu	☐	☐	☐	☐	☐
		Menu that considers the needs and preferences of all pupils	☐	☐	☐	☐	☐
		The opportunity to eat at school without pre-ordering	☐	☐	☐	☐	☐
	4.4 Are you involved in the school lunch / menu planning?	☐ Yes<br>☐ No					
	4.5. How are you involved in the school lunch / menu planning?						
	4.6 Would you be interested in the following activities and participation opportunities?		1 = Not interested	2	3	4	5 = Very interested
		Decorating the lunchroom	☐	☐	☐	☐	☐
		Giving feedback to the caterer	☐	☐	☐	☐	☐
		Voting for favorite meals	☐	☐	☐	☐	☐
		Test-tasting activities	☐	☐	☐	☐	☐

		Workshops on healthy and sustainable food	☐	☐	☐	☐	☐
		Participating in official school committees)	☐	☐	☐	☐	☐
		Involvement in a company run by pupils	☐	☐	☐	☐	☐
	4.7 What special requests or suggestions do you have for lunch at your school?						

5. We would like to know something about your general eating habits.	5.1 Are you currently following a special diet? Please select all answers that apply.	☐ Low-fat and low-calorie diet<br>☐ Low sugar diet<br>☐ Vegan diet<br>☐ Vegetarian diet<br>☐ Consumption of fish but no meat<br>☐ Lactose-free diet<br>☐ Gluten-free diet<br>☐ Halal diet<br>☐ Kosher diet<br>☐ No special diet<br>☐ Another diet:

			Daily (7 days)	Almost daily (5-6 days)	(3-4 days)	(1-2 days)	Less than 1-2 days	Never
	5.2 On how many days a week do you usually eat the following food at home?	Freshly cooked food	☐	☐	☐	☐	☐	☐
		Fast Food (e.g. Burger, Pizza, French fries)	☐	☐	☐	☐	☐	☐

		Vegetables or salad (no potatoes), Fruit	☐	☐	☐	☐	☐	☐
		Sweets or cake	☐	☐	☐	☐	☐	☐
		Salty snacks (e.g. potato or bread chips, salted pretzels or cheese sticks)	☐	☐	☐	☐	☐	☐
		Sweet drinks (Cola, lemonade, energy drinks or ice tea)	☐	☐	☐	☐	☐	☐
		Wholegrain products (e.g. wholegrain bread, pasta, or rice)	☐	☐	☐	☐	☐	☐

			1 = Does not apply	2	3	4	5 = Does apply
6. At the end of the survey, we would like to find out a little more about you, your school and family environment.	6.1 Please read the following statements carefully. To what extent do they apply on a scale of 1 (Does not apply) to 5 (Does apply)?	I like going to schools.	☐	☐	☐	☐	☐
		I feel comfortable in my class.	☐	☐	☐	☐	☐
		We have a good class community.	☐	☐	☐	☐	☐

		I have several good friends in my class.	☐	☐	☐	☐	☐
		I often meet up with some of my classmates after school.	☐	☐	☐	☐	☐
	6.2 How much do you trust the following people?		1 = No trust				5 = High trust
		Classmates	☐	☐	☐	☐	☐
		Teachers	☐	☐	☐	☐	☐
		Caterer at your school	☐	☐	☐	☐	☐
	6.3 Are you active in a sports club in your free time or do you volunteer for an organization that is committed to environmental protection, culture or social issues?	☐ Yes<br>☐ No					
	6.4 Are there times at home between Monday and Friday where all family members eat together?	☐ Yes<br>☐ No					

	6.5 What meals are these and on how many days do you eat them together with your family from Monday to Friday?		5 times per week)	3-4 times per week	1-2 times per week	Never
		Breakfast	☐	☐	☐	☐
		Afternoon lunch	☐	☐	☐	☐
		Dinner	☐	☐	☐	☐
	6.6 Here are some statements about eating habits in your family. How much do these statements apply to you and your family?		1 = Does not apply	2	3	4 = Does apply
		Having dinner together plays a big role in my family	☐	☐	☐	☐
		At home, everyone eats whenever they want.	☐	☐	☐	☐
		We have a dining table at home and enough seats for every family member.	☐	☐	☐	☐
		We only manage to eat together at home on weekends.	☐	☐	☐	☐
	6.7 Do you speak a language other than German at home?	☐ Yes<br>☐ No				
	6.8 What language do you speak at home other than German?					

	6.9 Which of the following statements apply to you and your family?		Yes	No
		I have my own room at home.	☐	☐
		Before Corona, my family and I went on vacation at least once a year.	☐	☐
		My cell phone/smartphone is never older than 2 years.	☐	☐
		I have my own computer/LapTop/tablet.	☐	☐
		I have my own games console at home.	☐	☐
	6.9 Do you currently have a valid "berlinpass-BuT"?	☐ Yes<br>☐ No<br>☐ I do not know		
	6.10 Do you use your "berlinpass-BuT" for a warm school lunch?)	☐ Yes<br>☐ No		

Supplementary Table S2 Characteristics of participating schools

**Size in number of pupils (8th-10th grade)**	**n**	**%**
Small (<500)	11	44
Medium (500-1000)	7	28
Large (>1000)	7	28
**Total**	25	100
**Districts**		
Mitte	2	8
Friedrichshain-Kreuzberg	2	8
Pankow	2	8
Charlottenburg-Wilmersdorf	5	20
Spandau	2	8
Steglitz-Zehlendorf	2	8
Tempelhof-Schöneberg	1	4
Neukölln	3	12
Treptow-Köpenick	1	4
Marzahn-Hellersdorf	0	0
Lichtenberg	2	8
Reinickendorf	3	12
**Total**	25	100
**All-day operations**		
with mandatory afternoon classes	22	88
without mandatory afternoon classes	3	12
**Total**	25	100

Supplementary Table S3 Characteristics of survey participants

**Gender**	**n**	**%**
Female	1353	45.2
Male	1532	51.2
Diverse	108	3.6
**Total**	2993	100
**Grade level**		
8th grade	577	19.3
9th grade	1227	40.9
10th grade	1193	39.8
**Total**	2997	100
**Birth year**		
2004	105	3.7
2005	511	18
2006	1072	37.7
2007	920	32.4
2008	233	8.2
**Total**	2841	100

Supplementary Table S4 COREQ Checklist for the qualitative research (Two networking events, three coaching sessions and one cooking workshop)

**Item No.**	**Guide Questions/Description**	**Reporting**
**Domain 1: Research team and reflexivity**		
Personal characteristics		
1. Interviewer/facilitator	Which authors participated in the events?	B.H. & M.S.
2. Credentials	What were the researcher's credentials? E.g. PhD, MD	B.H.: PhD<br>M.S.: habil.
3. Occupation	What was their occupation at the time of the study?	B.H.: Postdoc and researcher<br>M.S.: Managing director and principal investigator
4. Gender	Was the researcher male or female?	B.H.: male<br>M.S.: female
5. Experience and training	What experience or training did the researcher have?	Both researchers have advanced experiences in conducting qualitative interviews, focus groups and participatory observations
Relationship with participants		
6. Relationship established	Was a relationship established prior to study commencement?	For the school survey, contact was established to some participants prior to the events.
7. Participant knowledge of the interviewer	What did the participants know about the researcher? e.g. personal goals, reasons for doing the research	The research objectives were communicated to the participants in the invitation letters and at the start of the events.
8. Interviewer characteristics	What characteristics were reported about the inter viewer/facilitator? e.g. Bias, assumptions, reasons, and interests in the research topic	The researchers were interested in exploring the acceptance and quality of school meals in secondary schools in Berlin but also in related health and social cohesion implications.

**Domain 2: Study design**		
Theoretical framework		
9. Methodological orientation and Theory	What methodological orientation was stated to underpin the study? e.g. grounded theory, discourse analysis, ethnography, phenomenology, content analysis	The study was based on the interpretative paradigm of qualitative research. A combination of inductive and deductive coding was used for the analysis.
Participant selection		
10. Sampling	How were participants selected? e.g. purposive, convenience, consecutive, snowball	All schools that participated in the survey were invited to take part in the events. From these schools we aimed to invite all relevant stakeholders involved in the task of school catering (e.g. municipal school authorities, principals, teachers, caterers, parents and pupils)
11. Method of approach	How were participants approached? e.g. face-to-face, telephone, mail, email	The schools were approached via email by the Networking Center for School Catering (NCSC)
12. Sample size	How many participants were in the study?	First networking event: 8 participants; second networking event: 34 participants; first coaching session: 9 participants; second coaching session: 6 participants; third coaching session: 7 participants; cooking workshop: 5 participants
13. Non-participation	How many people refused to participate or dropped out? Reasons?	Most schools invited did not participate in the events due to high work load and limited capacities.
Setting		
14. Setting of data collection	Where was the data collected? e.g. home, clinic, workplace	Data was collected in a seminar room at the Center for Technology and Society as well as online (Networking events), in three schools and in a training kitchen of the "Kantine Zukunft Berlin".

15. Presence of non-participants	Was anyone else present besides the participants and researchers?	In the networking events and the coaching sessions, the NCSC gave input and moderated the follow-up discussion. The workshop was held by a professional cook. A representative of the NCSC was also present but did not actively participate.
16. Description of sample	What are the important characteristics of the sample? e.g. demographic data, date	Demographic data was not inquired. The participants were selected according to their function. For instance, principals, teachers, caterers, representatives from the municipal school authorities, pupils and parents
Data collection		
17. Interview guide	Were questions, prompts, guides provided by the authors? Was it pilot tested?	Interview guidelines were not used. The events were planned according to a program which was intended to give the participants the space for an open discussion.
18. Repeat interviews	Were repeat interviews carried out? If yes, how many?	No
19. Audio/visual recording	Did the research use audio or visual recording to collect the data?	No
20. Field notes	Were field notes made during and/or after the interview or focus group?	Researchers made field notes during the events. Meeting minutes were written afterwards.
21. Duration	What was the duration of the inter views or focus group?	First networking event: two hours; second networking event: three hours and thirty minutes; all coaching sessions: two hours; cooking workshop: eight hours and thirty minutes
22. Data saturation	Was data saturation discussed?	Data saturation was not discussed However, the validity of data collection was improved by discussing key findings with the NCSC. This association has many years of experiences in the field of Berlin school catering

23. Transcripts returned	Were transcripts returned to participants for comment and/or correction	Findings and drawn conclusions were discussed with the NCSC.
**Domain 3: analysis and findings**		
Data analysis		
Number of data coders	How many data coders coded the data?	The data was coded by B.H. The findings were later discussed together with M.S. and our practice partners.
Description of the coding tree	Did authors provide a description of the coding tree?	No
Derivation of themes	Were themes identified in advance or derived from the data?	Themes were derived from the data in an inductive manner.
Software	What software, if applicable, was used to manage the data?	No software was used for the coding
Participant checking	Did participants provide feedback on the findings?	Findings were discussed with our practice partners.
Reporting		
Quotations presented	Were participant quotations presented to illustrate the themes/findings? Was each quotation identified? e.g. participant number	Main findings are derived from the minutes of events. Quotations are identified by participant number and event.
Data and findings consistent	Was there consistency between the data presented and the findings?	Findings were supported by key themes.
Clarity of major themes	Were major themes clearly presented in the findings?	Key themes are presented in the findings and discussion section.
Clarity of minor themes	Is there a description of diverse cases or discussion of minor themes?	Due to the article length and format requirements, minor themes could not be included in the findings.

Source: Tong A, Sainsbury P, Craig J. Consolidated criteria for reporting qualitative research (COREQ): a 32-item checklist for interviews and focus groups. *International Journal for Quality in Health Care.* **2007**. 19 (6), 349 – 357.

Supplementary Table S5 Frequency of participation in warm school lunch (if available)

How often do you eat warm lunch at school	Never	Less often	1-2 times per week	3-4 times per week	Daily	Total
n	1742	321	139	165	243	2610
%	66.7	12.3	5.3	6.3	9.3	100

Supplementary Table S6 Reasons for never or rarely eating a warm lunch at school (multiple answers)

Why do you never or rarely eat at school? Please select all answers that apply (n = 2644)	n	%
Because I prefer to eat dinner at home.	611	23.1
Because I do not like the taste of the food.	585	22.1
Because the price is too high for me and my parents.	320	12.1
Because the lunchbreak is too short.	491	18.6
Because I prefer to eat with my friends outside of school.	260	9.8
Because I prefer to buy something from the kiosk/vending machine.	402	15.2
Because the lunchroom is not appealing.	208	7.9
Because of little variety in food/small menu.	235	8.9
Because the food contains too many calories and fat.	99	3.7
Because my friends do not eat at school.	342	12.9
Because I bring my food from home.	568	21.5
Because I feel overly supervised by teachers.	215	8.1
Because the food does not fit into my personal diet.	158	6
Because I am not particularly hungry at noon.	355	13.4
Because the portion size is too small	173	6.5
Because the portion size is too large	10	.3

Supplementary Table S7 Satisfaction with the food and the lunchroom

**How satisfied are you with the following aspects of the school lunch?**	**1 = not satisfied**	**2**	**3**	**4**	**5 = very satisfied**	**Total**
**Taste**						
n	95	164	284	225	97	865
%	11	19	32.8	26	11.2	100
**Temperature**						
n	87	158	252	241	128	866
%	10	18.2	29.1	27.8	14.8	100
**Food presentation**						
n	97	198	285	196	88	864
%	11.2	22.9	33	22.7	10.2	100
**Menu**						
n	141	212	263	164	87	867
%	16.3	24.5	30.3	18.9	10	100
**Length of lunch break**						
n	229	152	222	135	123	861
%	26.6	17.7	25.8	15.7	14.3	100
**Meal ordering system**						
n	110	162	253	197	134	856
%	12.9	18.9	29.6	23	15.7	100
**Service workers**						
n	91	119	183	188	281	862
%	10.6	13.8	21.2	21.8	32.6	100
**Noise level**						
n	202	217	259	135	49	862
%	23.4	25.2	30	15.7	5.7	100
**Available seating**						
n	134	202	268	162	95	861
%	15.6	23.5	31.1	18.8	11	100
**Cleanliness**						
n	124	183	270	188	98	863
%	14.4	21.2	31.3	21.8	11.4	100
**Comfort**						
n	152	222	288	126	70	858
%	17.7	25.9	33.6	14.7	8.2	100

Supplementary Table S8 Eating warm school lunch

Which of the following statements apply to you eating school lunch?	Disagree	Rather disagree	Rather agree	Agree	Total
**We eat lunch together with the class**					
n	556	146	107	88	897
%	62	16.3	11.9	9.8	100
**I only eat lunch with my friends**					
n	134	109	272	384	899
%	14.9	12.1	30.3	42.7	100
**The school lunch is an opportunity for me to talk to other pupils that I do not know so well.**					
n	322	271	209	91	893
%	36.1	30.3	23.4	10.2	100

Supplementary Table S9 Opportunities to participate in school lunch

Are you involved in the school lunch / menu planning?	Yes	No	Total
n	156	2326	2482
%	6.3	93.7	100

Supplementary Table S10 Pupils' interests in participation opportunities

Would you be interested in the following activities and participation opportunities?	1 = Not interested	2	3	4	5 = Very interested	Total
**Decorating the lunchroom**						
n	610	407	795	372	348	2532
%	24.1	16.1	31.4	14.7	13.7	100
**Giving feedback to the caterer**						
n	537	359	829	427	358	2510
%	21.4	14.3	33	17	14.3	100
**Voting for favorite meals**						
n	459	302	632	473	646	2512
%	18.3	12.0	25.2	18.8	25.7	100
**Test-tasting activities**						
n	658	421	746	331	354	2510
%	26.2	16.8	29.7	13.2	14.1	100
**Workshops on healthy and sustainable food**						
n	902	537	630	244	209	2522
%	35.8	21.3	25	9.7	8.3	100
**Participating in official school committees**						
n	584	433	818	388	294	2517
%	23.2	17.2	32.5	15.4	11.7	100
**Involvement in a company run by pupils**						
n	761	518	783	269	176	2507
%	30.4	20.7	31.2	10.7	7	100

Supplementary Table S11 Trust in the caterer

How much do you trust your caterer at school?	1 = No trust	2	3	4	5 = High trust	Total
n	882	505	642	274	179	2482
%	35.5	20.3	25.9	11	7.2	100

Supplementary Table S12"berlinpass-BuT"

Do you currently have a valid "berlinpass-BuT"?	Yes	No	Total
n	707	1196	1903
%	37.2	62.8	100
**Do you use your "berlinpass-BuT" for a warm school lunch?)**	**Yes**	**No**	**Total**
n	281	418	699
%	40.2	59.8	100
